# H_2_ inhalation therapy in patients with moderate COVID-19 (H_2_COVID): a prospective ascending-dose phase I clinical trial

**DOI:** 10.1128/aac.00573-24

**Published:** 2024-07-17

**Authors:** C. Salomez-Ihl, J. Giai, M. Barbado, A. Paris, S. Touati, J. P. Alcaraz, S. Tanguy, C. Leroy, A. Lehmann, B. Degano, M. Gavard, P. Bedouch, P. Pavese, A. Moreau-Gaudry, M. Roustit, F. Boucher, P. Cinquin, J. P. Brion

**Affiliations:** 1 Université Grenoble Alpes, CNRS, UMR 5525, VetAgro Sup, Grenoble INP, CHU Grenoble Alpes, TIMC, UMR5525, Grenoble, France; 2 Department of Pharmacy, Université Grenoble Alpes, CHU Grenoble Alpes, Grenoble, France; 3 Univ. Grenoble Alpes, Inserm, CHU Grenoble Alpes, Centre for Clinical Investigation, Grenoble, France; 4 Department of Infectious and Tropical Diseases, CHU Grenoble Alpes, Grenoble, France; 5 Department of Pneumology, CHU Grenoble Alpes, Grenoble, France; 6 CHU Grenoble Alpes, Delegation for Clinical Research and Innovation, Grenoble, France; IrsiCaixa Institut de Recerca de la Sida, Barcelona, Spain

**Keywords:** COVID-19, molecular hydrogen, administration, inhalation

## Abstract

The coronavirus disease 2019 (COVID-19) pandemic, caused by the severe acute respiratory syndrome coronavirus 2 (SARS-CoV-2), has triggered a serious global health crisis, resulting in millions of reported deaths since its initial identification in China in November 2019. The global disparities in immunization access emphasize the urgent need for ongoing research into therapeutic interventions. This study focuses on the potential use of molecular dihydrogen (H2) inhalation as an adjunctive treatment for COVID-19. H2 therapy shows promise in inhibiting intracellular signaling pathways associated with inflammation, particularly when administered early in conjunction with nasal oxygen therapy. This phase I study, characterized by an open-label, prospective, monocentric, and single ascending-dose design, seeks to assess the safety and tolerability of the procedure in individuals with confirmed SARS-CoV-2 infection. Employing a 3 + 3 design, the study includes three exposure durations (target durations): 1 day (D1), 3 days (D2), and 6 days (D3). We concluded that the maximum tolerated duration is at least 3 days. Every patient showed clinical improvement and excellent tolerance to H2 therapy. To the best of our knowledge, this phase I clinical trial is the first to establish the safety of inhaling a mixture of H2 (3.6%) and N2 (96.4%) in hospitalized COVID-19 patients. The original device and method employed ensure the absence of explosion risk. The encouraging outcomes observed in the 12 patients included in the study justify further exploration through larger, controlled clinical trials.

**CLINICAL TRIALS:**

This study is registered with ClinicalTrials.gov as NCT04633980.

## INTRODUCTION

Severe acute respiratory syndrome coronavirus 2 (SARS-CoV-2) has been responsible for coronavirus disease 2019 (COVID-19) since November 2019, when it was first discovered in China. Since then, more than 770 million cases and almost 7 million deaths have been reported around the world (1). This pathology comes with life-threatening respiratory symptoms in severe cases, especially in patients with risk factors such as age, obesity, diabetes, and cardiovascular diseases (2). Since the appearance of this pathology, epidemiological data have evolved, thanks to the development of vaccination. Indeed, the vaccine protects against severe forms and has contributed to a significant reduction in hospitalizations and deaths (3). Nevertheless, access to immunization shows significant disparities across the world (4). Therefore, it remains essential to continue the research effort for therapeutic strategies.

In this context, dihydrogen (H_2_) inhalation could be an interesting opportunity. Hyperbaric H_2_ inhalation was first described in the 1970s (5) to have potential for cancer treatment, and the first preclinical study at atmospheric pressure dates back to 2007 (6), in a model of cerebral infarction in the rat. Since then, Ito et al. (7) have shown that H_2_ inhibits intracellular signaling pathways of inflammation without involving anti-free radical effects. In addition, H_2_ inhalation (2.9%) has also been reported to limit mast cell activation (8). Xie et al. have shown that two 60-min sessions of inhalation of a gas mixture containing 2% H_2_ allow limitation of multiple-organ damage and mortality in a model of generalized inflammation in mice (9). They also have shown that inhaling H_2_ restores the PaO_2_:FiO_2_ ratio, both in a mouse model of sepsis by cecal ligation (10) and in a model of lung damage induced by lipopolysaccharides (11). H_2_ has also been described as reducing the significant burden on lung parenchyma during COVID-19 (12). In view of the current data in the literature, the application of a H_2_ treatment makes it possible to trigger numerous potentially protective mechanisms in a hyperinflammatory context, such as sepsis and very probably COVID-19, by trapping hydroxyl radicals and peroxynitrite, by limiting inflammatory reactions by modulating intracellular transduction cascades, and by modifying the expression of certain genes (13).

About tolerance, H_2_ has been safely used in the 1960s at very high doses, to prevent decompression sickness and arterial gas thrombi, in deep-diving gas mixes (hydreliox = breathing gas mixture used at high pressure—60 bars—containing 49% H_2_, 50% helium, and 1% O_2_) (14). In the clinical context, H_2_ has been shown to have no effect on temperature, blood pressure, pH, or peripheral capillary oxygen saturation (SpO_2_) (15). In humans, no adverse effects related to H_2_ have been described with H_2_ inhalation in hundreds of patients until now (16).

Concerning administration, several routes have been considered. The most widely used today, both in preclinical and clinical trials, are the ingestion of hydrogen-enriched drinking water and the inhalation of gas mixtures (17). H_2_ is considered highly flammable when its concentration in the air exceeds 4.1% (18). As a result, until recently, the used gas mixtures all contained between 2% and 4% H_2_.

In Spring 2020, when we initiated this research, anti-inflammatory strategies such as corticosteroids were the only medications that showed efficacy in COVID-19 patients. Chinese guidelines already recommended the use of H_2_ in COVID-19 patient management (19, 20). Then, a Chinese team published results of using H_2_ in 2020 in an efficacy open label clinical trial with the administration of a mixture including 67% H_2_ and 33% of O_2_, with statistically significant improvement of clinical and biological parameters (21). Since then, other studies using inhalation of mixtures with 66% of H_2_ in acute and post-acute COVID-19 have been launched (22, 23). However, the explosion hazard is not discussed, and these mixtures do not comply with norms and regulations in several countries. Our hypothesis is that the administration of H_2_ mixtures below the explosivity level of 4.1% could safely improve the clinical condition of hospitalized patients with moderate COVID-19 [World Health Organization (WHO) clinical progression scale score of 5 (24)]. The primary aim of this study was to establish the feasibility and safety of an original protocol of H_2_ inhalation by defining its maximum tolerated duration (MTD).

## MATERIALS AND METHODS

### Study design

**Fig 1:**
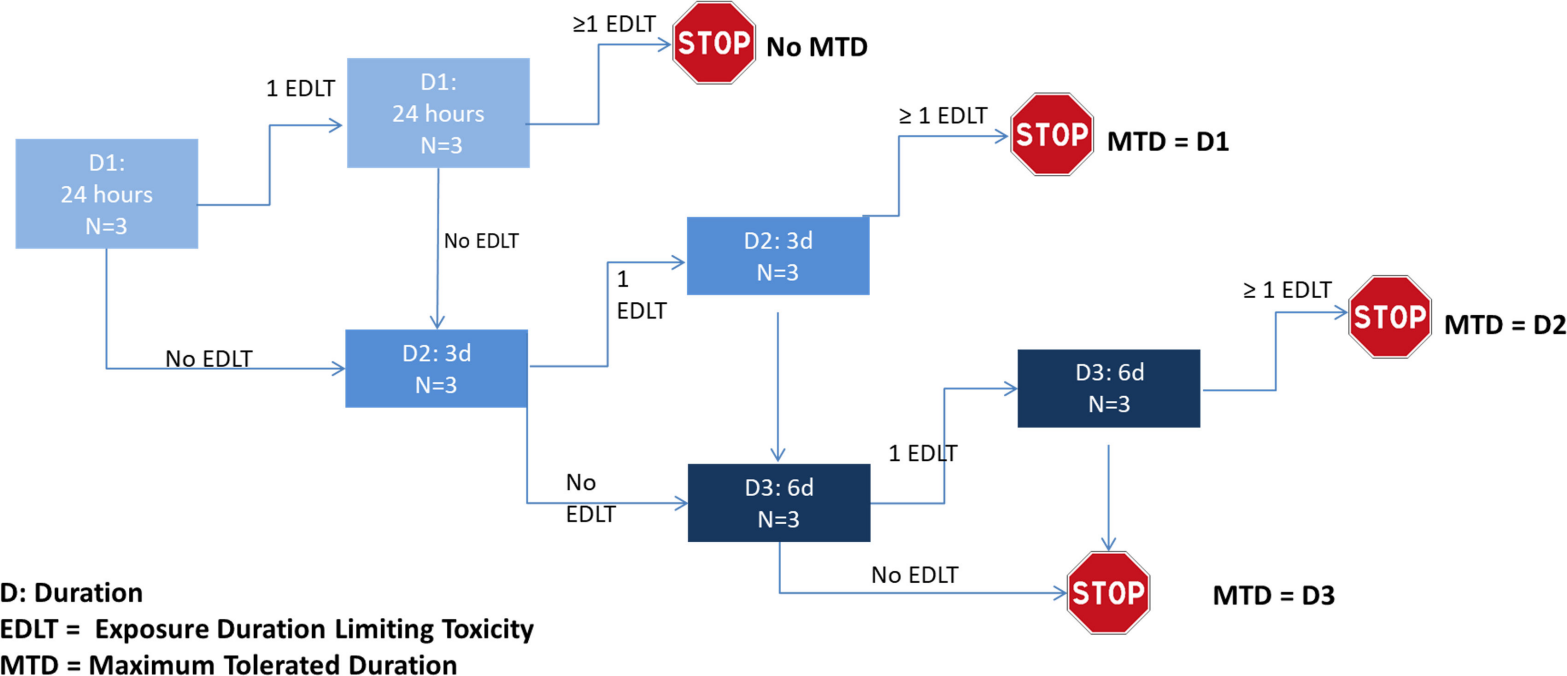
Design of the Single Ascending Dose study conducted in patients, with three doses (target durations) tested (D1–D3). D, duration; EDLT, exposure duration-limiting toxicity; MTD, maximum tolerated duration.

This phase I, open-label, prospective, monocentric, single ascending-dose study aims to establish the safety and the tolerability of the procedure in patients with confirmed SARS-CoV-2 infection. A 3 + 3 design was used, with three durations of exposure (target durations): 1 day (D1), 3 days (D2), and 6 days (D3), as summarized in Fig. 1 and in the supplemental material.

### Study population

We included adult patients with suspicion of SARS CoV-2 infection based on clinical signs and positive PCR, and hospitalized with SpO_2_ ≤94% on room air requiring normobaric oxygen therapy with a nasal flow of O_2_ ≤ 6 L /min to reach at least SaO_2_ ≥95%. Detailed inclusion and exclusion criteria are specified in the supplemental material.

### Interventions

All patients received the usual standard of care during their hospitalization (antibiotics, systemic corticosteroid therapy, and preventive anti-coagulation).

**Fig 2:**
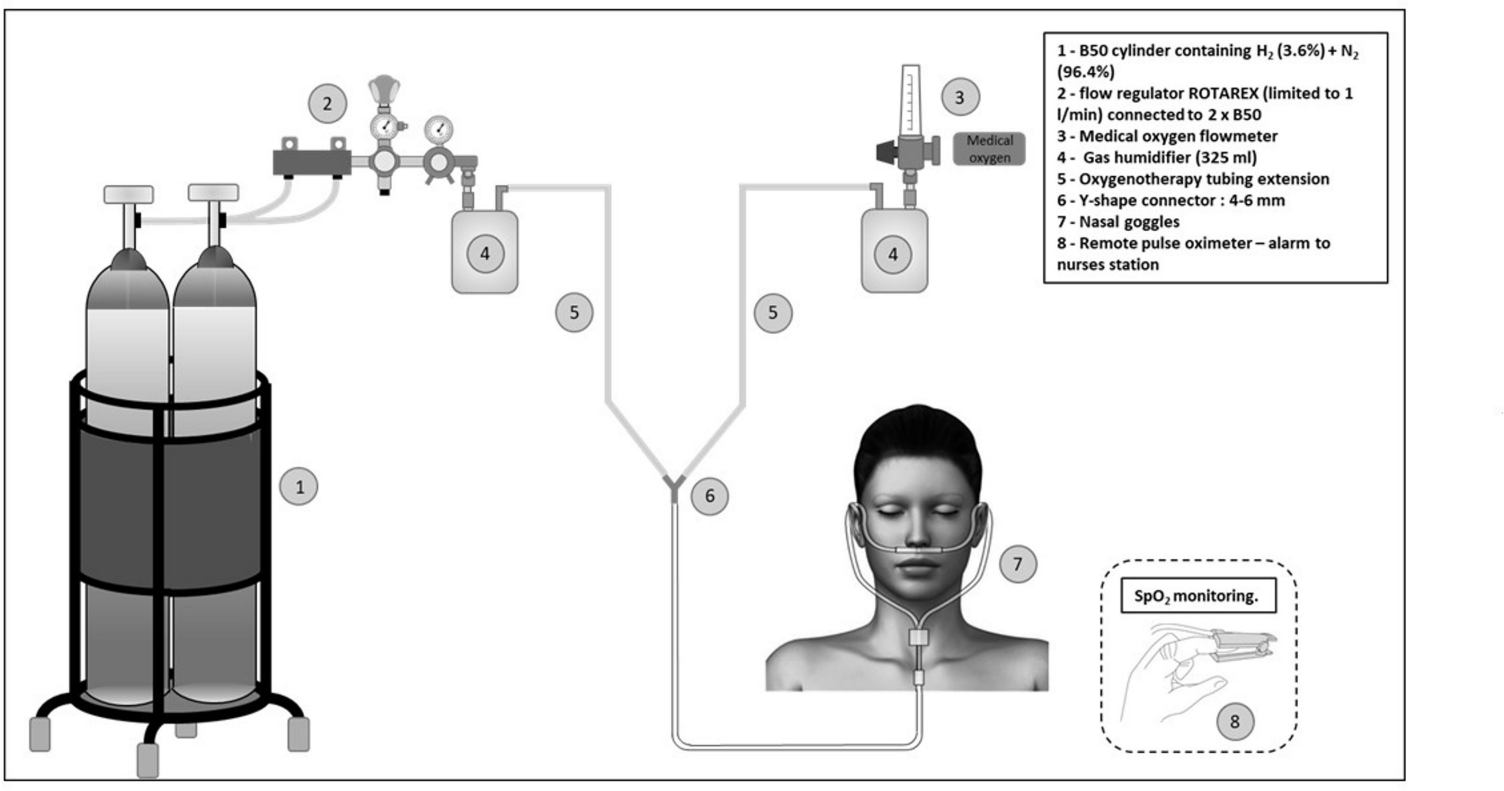
Overview of hydrogen inhalation in COVID-19 patients.

An original medical delivery device has been designed by our team and has undergone a risk analysis by an independent organization. This device includes a flow regulator (a CE-marked medical device for clinical trials) allowing guarantee of a fixed flow of 1 L/min of a specific medical-grade gas mixture (3.6% H_2_ and 96.4% N_2_), manufactured and supplied by Air Products, packaged in B50-type cylinders. The gas mixture is combined with O_2_ from the oxygen outlet of the wall (O_2_ flow adapted to the needs of the patient, in accordance to standard of care). The device and method ensure that there is no risk of explosion or ignition. The medical delivery device is illustrated in Fig. 2. If a clinical and radiological improvement occurred, and if SpO_2_ remained above 95%, O_2_ and H_2_ inhalations were stopped even if the target duration of 24 h and 3 or 6 days was not achieved.

Specific training has been given to user personnel, and information has been provided to guarantee suitability for use (posters and explanatory documents).

### Outcomes

Because the concentration and the flow of the inhaled mixture are kept constant, the term “dose” actually refers to the target duration of exposure to H_2_ inhalation. The classical notion of dose-limiting toxicity therefore becomes “exposure duration-limiting toxicity” (EDLT) and is defined as the occurrence of any of the following serious adverse events (SAEs) rated according to the National Institutes of Health Common Terminology Criteria for Adverse Events (CTCAE v.5.0) (25) during and over 3 days after the end of H_2_:

- Observed grade of ≥4 toxicity from the Respiratory, Thoracic and Mediastinal Disorders section of CTCAE v.5.0.
- Observed grade of ≥3 toxicity from other sections of CTCAE (v.5.0).
- Any relevant deterioration in the health of the subject.
- At least possibly related with H_2_.

The primary outcome of this study is the MTD, defined as the maximum duration of exposure to H_2_ with no more than one EDLT. If an EDLT occurs, three additional patients without EDLT have to be included to authorize moving to the next step. As a consequence, between 6 and 24 patients could have been included in the study (see Table 1; Fig. 1).

**TABLE 1:** Population description

Cohort	1-day treatment (*n* = 3)	3-day treatment (*n* = 3)	6-day treatment (*n* = 6)	Total (*N* = 12)
Age				
Mean (SD)	52.67 (6.66)	56.33 (1.53)	62.33 (14.11)	58.42 (10.84)
Median	56.00	56.00	64.50	57.00
Q1–Q3	50.50–56.50	55.50–57.00	57.00–72.75	55.75–61.50
Min–max	45.00–57.00	55.00–58.00	39.00–76.00	39.00–76.00
*n*	3	3	6	12
Sex, *n* (col %)				
Female	1 (33.3)	0 (0.0)	0 (0.0)	1 (8.3)
Male	2 (66.7)	3 (100.0)	6 (100.0)	11 (91.7)
*n*	3	3	6	12
Weight (kg)				
Mean (SD)	88.00 (8.19)	77.33 (11.15)	77.50 (16.13)	80.08 (13.26)
Median	90.00	73.00	77.50	81.00
Q1–Q3	84.50–92.50	71.00–81.50	63.75–86.75	71.25–90.00
Min–max	79.00–95.00	69.00–90.00	60.00–101.00	60.00–101.00
*n*	3	3	6	12
Height (cm)				
Mean (SD)	173.00 (11.36)	177.33 (3.21)	176.50 (5.96)	175.83 (6.67)
Median	178.00	176.00	177.50	177.50
Q1–Q3	169.00–179.50	175.50–178.50	172.50–181.00	174.00–181.00
Min–max	160.00–181.00	175.00–181.00	168.00–183.00	160.00–183.00
*n*	3	3	6	12
Body mass index (kg/m^2^)				
Mean (SD)	29.67 (5.03)	24.47 (2.66)	24.68 (3.89)	25.88 (4.25)
Median	29.00	23.80	23.75	24.90
Q1–Q3	27.00–32.00	23.00–25.60	21.88–27.20	22.58–28.25
Min–max	25.00–35.00	22.20–27.40	20.50–30.50	20.50–35.00
*n*	3	3	6	12

An independent clinical events committee (CEC) was constituted: (i) to review all adverse events before each duration increase; (ii) each time an SAE occurred, in order to assess the imputability of H_2_; (iii) on investigator or sponsor demand. The study had to be discontinued anytime on CEC request, particularly if an SAE was attributed to the intervention, which would have immediately stopped the study.

### Statistical analysis

The study population was the intention to treat population, i.e., patients were analyzed in the initial cohort they were allocated to, even if H_2_ therapy was stopped prematurely. Descriptive statistics were performed for all evaluation criteria. Categorical variables were presented using counts and frequencies, while continuous variables were presented using mean, standard deviation, minimum, median, maximum, interquartile range, and number of subjects with evaluable data. Normality of continuous variables was assessed graphically. Analysis was performed with R software (v.≥4.2).

### Role of the funding source

The funders of the study had no role in the study design, data collection, data analysis, data interpretation, writing of the report, or decision to submit for publication. The authors take responsibility for and guarantee the integrity and completeness of the data, the accuracy of the data analysis and the fidelity to the protocol which they supervised at every stage.

## RESULTS

### Description of population

Twelve participants were recruited from the Department of Infectious Diseases at the University Hospital of Grenoble, from 19 January 2021 to 31 May 2022. The last patient follow-up was 7 June 2022. Three received 1 day of treatment and three received 3 days of treatment. Six patients were recruited for the 6-day treatment. Among these patients, two were treated for the entire planned duration and four prematurely discontinued the treatment because their clinical improvement was such that they no longer required oxygen therapy and were discharged from the hospital.

Overall, 11 patients were male (92%); the mean age was 58 (SD = 10.8); and the mean body mass index was 26 (SD = 4.2).

Patient data at trial entry are available in Table 2.

**TABLE 2:** Patient characteristics

Patient	Comorbidities	Vaccination status	On patient admission			On patient inclusion			
			Oxygen partial pressure (mmHg)	Carbon dioxide partial pressure (mmHg)	Oxygen saturation (%)	Oxygen partial pressure (mmHg)	Carbon dioxide partial pressure (mmHg)	Oxygen saturation (%)	Concomitant treatments associated with the management of COVID-19
1	High blood pressure Type 2 diabetes	Not vaccinated	90.3	35.3	93	Same sample as at admission	Same sample as at admission	99	O_2_ 2 L/min Amoxicillin (PO) Prednisolone (PO) 60 mg Enoxaparin sodium (subcutaneous) 4,000 UI
2	No	Not vaccinated	98.4	39.9	98	142.9	33.6	99	O_2_ 2 L/min Enoxaparin sodium (subcutaneous) 4,000 UI Salmeterol (spray) 0.25/0.05 mg Prednisolone (PO) 60 mg
**3**	High blood pressure Dyslipidemia	Not vaccinated	69.6	32.6	95	Same sample as at admission	Same sample as at admission	96	O_2_ 4 L/min Enoxaparin sodium (subcutaneous) 4,000 UI Salmeterol (spray) 0.25/0.05 mg Prednisolone (PO) 50 mg
4	No	Not vaccinated	Not measured	Non réalisé	92	63.2	32.5	94	O_2_ 3 L/min Enoxaparin sodium (subcutaneous) 4,000 UI Prednisolone (PO) 40 mg
5	No	Not vaccinated	97.2	32.4	97	Same sample as at admission	Same sample as at admission	94	O_2_ 2 L/min Rivaroxaban (PO) 15 mg twice a day Prednisolone (PO) 50 mg
6	High blood pressure	Not vaccinated	69.8	36.2	93	75.4	38.7	97	O_2_ 3 L/min Enoxaparin sodium (subcutaneous) 4,000 UI Prednisolone (PO) 50 mg
7	No	Not vaccinated	Not measured	Not measured	94	78.2	36.4	98	O_2_ 4 L/min Enoxaparin sodium (subcutaneous) 4,000 UI Prednisolone (*PO*) 60 mg
8	No	Vaccinated one dose	65	30.5	92	Non-interpretable sample	Non-interpretable sample	99	O_2_ 4 L/min Enoxaparin sodium (subcutaneous) 4,000 UI Prednisolone (*PO*) 50 mg
9	Wegener’s disease Chronic back pain following a workplace accident Chronic hypoxemia with negative jak2 mutation Obstructive sleep apnea syndrome	Vaccinated two doses	95.6	27.7	97	Same sample as at admission	Same sample as at admission	98	O_2_ 4 L/min Enoxaparin sodium (subcutaneous) 4,000 UI
10	Nodular basal cell carcinoma at the junction between the nose and cheek	Vaccinated two doses	104.3	30.1	92	Same sample as at admission	Same sample as at admission	94	O_2_ 4 L/min Enoxaparin sodium (subcutaneous) 4,000 UI Prednisolone (PO) 40 mg
11	Lymphoma in remission, which started in 2019	Vaccinated three doses	71.1	30.2	96	91.1	30.5	97	O_2_ 1 L/min Dexaméthasone (PO) 6 mg Enoxaparin sodium (subcutaneous) 4,000 UI
12	Lung adenocarcinoma diagnosed in September 2021, multi-metastatic in liver, bone, and brain Mesenteric ischemia in 2006 with surgical management and diagnosis of coagulopathy due to excess factor VIII on long-term anti-coagulation High blood pressure Dyslipidemia	Vaccinated four doses	69.5	43.1	91	75.4	45	93	O_2_ 2 L/min Enoxaparin sodium (subcutaneous) 6,000 UI twice a day

A CT scan (iodin injection was done if pulmonary embolism was suspected) was performed at inclusion for all patients except two. In all cases, he CT scan showed specific images of COVID-19 pneumonia, and the proportion of damage was found between 25% and 50%.

On average, patients were included 12.2 days after the onset of their symptoms (SD = 2.0) (minimum: 9 days, maximum: 16 days).

### Primary outcome

The maximum tolerated duration was at least 3 days, since only two out of the six patients included in step 3 of the study (D3, 6-day treatment) were treated for 6 days. Indeed, the clinical condition of the other four patients included improved so well before D3 that discontinuation of oxygen therapy was decided before the end of this period, so that this step could not be validated. Two SAEs occurred. The first one occurred in a patient whose oxygen requirement increased during the 3-day observation period after the end of H_2_ inhalation. This patient was admitted during 24 h in intensive care unit, received up to 40-L/min of O_2_ supplement, and rapidly recovered. The second SAE occurred in a patient with a pulmonary embolism, which did not preclude the continuation of the H_2_ therapy. Both SAEs were attributed to COVID-19 and not to H_2_ by the CEC.

Changes in patients’ biological and clinical variables are shown in the supplemental material.

## DISCUSSION

All patients who improved clinically tolerated H_2_ therapy perfectly. This study is, to our knowledge, the first phase I clinical trial to demonstrate the safety of the inhalation of a H_2_ (3.6%)-N_2_ (96.4%) mixture in hospitalized COVID-19 patients, with an original device and method guaranteeing the absence of explosion risk. We demonstrated an MTD of at least 3 days, which seems to be sufficient for the management of patients with COVID-19 with a WHO scale score of 5, since clinical improvement is significant at day 3.

Our results remain very encouraging concerning longer durations of treatment, since none of the six patients treated in the D3 group of our study presented any adverse effects attributed to H_2_. All observed adverse events are complications of moderate COVID-19, well described in the literature (26, 27).

The methodology of a phase I test does not allow conclusion on the possible efficacy of the gas mixture. However, results are very encouraging, as described in the supplemental material.

H_2_ has been described as having the ability to reduce lung injury and thus to reduce the number of critically ill patients (28). Another literature review has explained that H_2_ could directly enter the lung tissue through ventilatory activities and exert anti-inflammatory effects at the multiple stages of the inflammatory response, alleviating the airway damage caused by the excessive activation of the inflammatory cells and the massive release of inflammatory factors (29). In addition, during COVID-19-associated pulmonary injury, activation of resident alveolar macrophages has led to the release of potent proinflammatory mediators and chemokines that promote the accumulation of neutrophils and monocytes (30). Inhaled H_2_ exerts a non-specific anti-inflammatory effect on macrophages, neutrophils, and lymphocytes and inhibits reactive oxygen species production (31).

The designed delivery device guaranteed a fixed flow rate of 1 L/min of the gas mixture while allowing the adaptation of the O_2_ flow rate to the patient’s needs. Since this flow is completed by the patient’s natural breathing, there is no risk of suffocation of the patient, even if the device is improperly used. At a concentration of 3.6% of H_2_ in the mixture, the patient receives 1.5 mmol/min 24 h a day, corresponding to 2,160 mmol/day. This dose is significantly lower than the dose administered in the protocols where the patient inhales a stoichiometric mixture of H_2_ 66%-O_2_ 33%. However, the question of the explosion hazard associated with the clinical use of this stoichiometric mixture is not addressed in the corresponding publications. Literature data (15) have led us to suggest that much lower concentrations, respecting the safety standards accepted in the majority of countries, have an anti-inflammatory activity and therefore could have an efficacy against COVID-19 comparable to that reported in China.

Finally, demonstrating the safety of an H_2_ inhalation protocol compatible with explosion risk standards opens up the possibility of ambulatory use of H_2_ gas. Beyond COVID-19, there is a considerable potential for the combined ambulatory administration of O_2_ and H_2_ to the lungs. Indeed, H_2_ is the only known molecule with anti-inflammatory properties that is totally devoid of recognized adverse effects.

### Conclusion

We demonstrated that H_2_ inhalation at 3.6% delivered with our device is a safe therapy in humans, including those with viral pulmonary pathology. This clinical trial is the first step toward approval of our H_2_ inhalation protocol as a drug delivered by a medical device. More data are obviously awaited. In particular, it would be important to carry out phase II and III clinical trials, with a much larger number of patients, in order to demonstrate H_2_ efficacy in the management of pathologies involving oxidative and inflammatory phenomena, including of course COVID-19. Pathologies with strong pulmonary inflammatory component, such as chronic obstructive pulmonary disease, could also benefit greatly from this potential therapy.

## SUPPLEMENTAL MATERIAL

Supplemental figures aac.00573-24-s0001.docx Figures S1 and S2.

ASM does not own the copyrights to Supplemental Material that may be linked to, or accessed through, an article. The authors have granted ASM a non-exclusive, world-wide license to publish the Supplemental Material files. Please contact the corresponding author directly for reuse.
