# Supplementary material for: H2 inhalation therapy in patients with moderate COVID-19 (H2COVID): a prospective ascending-dose phase I clinical trial
Source: Antimicrob Agents Chemother. 2024 Jul 17;68(8):e00573-24. doi: 10.1128/aac.00573-24 (PMC11304737; doi:10.1128/aac.00573-24)
Supplement: Supplemental figures — Figures S1 and S2. [file aac.00573-24-s0001.docx]

Supplemental data


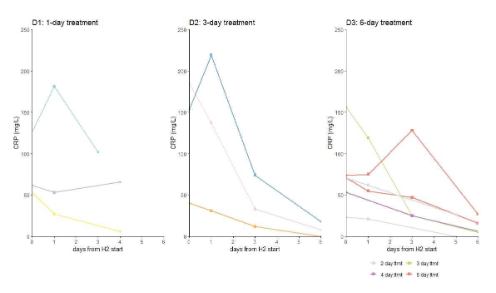


**Figure 1.** CRP evolution (mg/L) according to the number of days of treatment


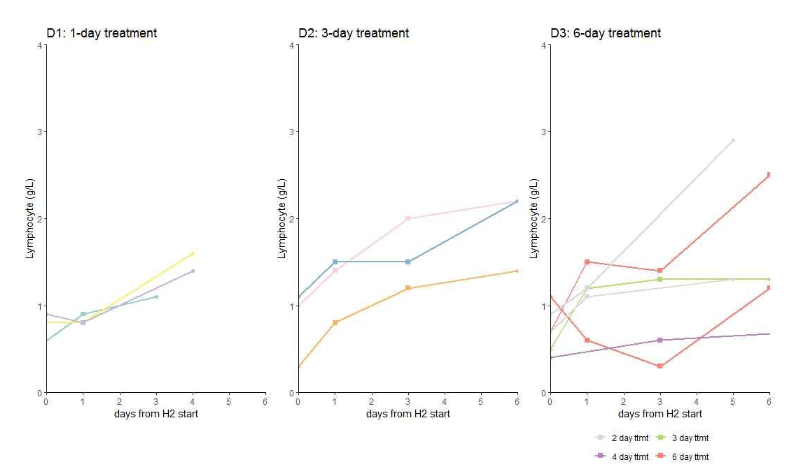


**Figure 2.** Lymphocytes evolution (G/L) according to the number of days of treatment
